# A 5-Month-Old Infant With a Complicated Holiday Souvenir

**DOI:** 10.1093/cid/ciae355

**Published:** 2024-12-17

**Authors:** Iris Voogd, Sanne A (S ) E Kooistra, Jamie A van der Meer, Soëba Ehsary, Clementien Vermont, Els van Nood, Marjolijn S W Quaak

**Affiliations:** Department of Internal Medicine, Erasmus MC University Medical Center, Rotterdam, The Netherlands; Department of Internal Medicine, Erasmus MC University Medical Center, Rotterdam, The Netherlands; Department of Internal Medicine, Erasmus MC University Medical Center, Rotterdam, The Netherlands; Department of Medical Microbiology and Infectious Diseases, Erasmus MC University Medical Center, Rotterdam, The Netherlands; Department of Pediatrics, Division of Infectious Diseases and Immunology, Erasmus MC University Medical Center–Sophia Children’s Hospital, Rotterdam, The Netherlands; Department of Internal Medicine, Erasmus MC University Medical Center, Rotterdam, The Netherlands; Department of Medical Microbiology and Infectious Diseases, Erasmus MC University Medical Center, Rotterdam, The Netherlands; Department of Pediatrics, Division of Infectious Diseases and Immunology, Erasmus MC University Medical Center–Sophia Children’s Hospital, Rotterdam, The Netherlands

##  

A 5-month-old Dutch male infant from non-consanguineous parents was referred to an academic children's hospital in the Netherlands because of a fever of unknown origin for 2 weeks and progressive pancytopenia. His previous medical history was unremarkable. He had been adequately vaccinated for his age. The travel history included a visit to Morocco at the age of 2 months.

Prior to presentation in our hospital, empirical sepsis treatment was initiated. However, blood cultures were negative and the child remained febrile after 48 hours of antibiotic treatment.

On referral, the child was hemodynamically stable but pale, inactive, and mildly dehydrated on physical examination. His body temperature was 39.6°C. Further physical examination revealed an enlarged spleen but was otherwise unremarkable. Splenomegaly was confirmed by ultrasound showing a total spleen size of 8 cm (suggested upper limit value at age 3–6 months: 6.5 cm) [1]. Laboratory tests revealed white blood cell counts of 2.4 × 10⁹/L (reference [ref]: 6.0–15.0 × 10⁹/L), hemoglobin of 7.9 g/dL (ref: 9.02–12.73 g/dL), platelet count of 67 × 10⁹/L (ref: 229–597 × 10⁹/L), C-reactive protein of 10 mg/dL (ref: <1 mg/dL), ferritin of 4662 µg/L (ref: 30–240 µg/L), lactate dehydrogenase of 1053 U/L (ref: <610 U/L), aspartate aminotransferase of 154 U/L (ref: 89 U/L), and alanine transaminase of 43 U/L (ref: <60 U/L). A serum polymerase chain reaction (PCR) test for cytomegalovirus (CMV) and the Epstein-Barr virus (EBV) were negative. A bone marrow biopsy was performed. The microscopy is shown in Figure 1.

**Figure 1.**
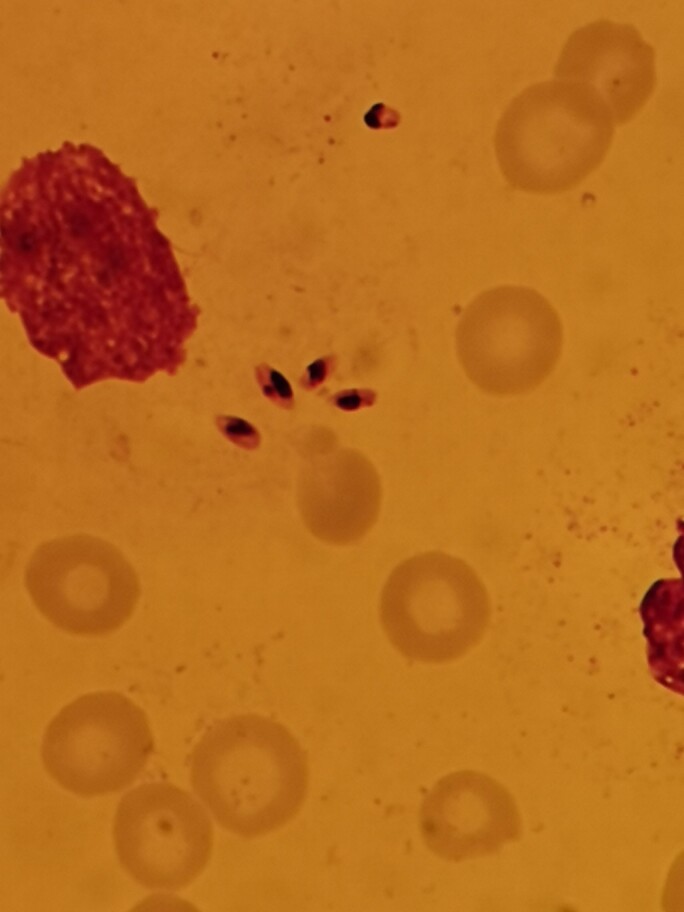
Giemsa staining of the bone marrow biopsy specimen.

What is your diagnosis?

##  

Diagnosis: Visceral leishmaniasis, with suspicion of hemophagocytic lymphohistiocytosis

The presentation with prolonged fever, pancytopenia, and elevated inflammatory markers was suggestive for a hyperinflammatory syndrome. Since 6 of 8 diagnostic hemophagocytic lymphohistiocytosis (HLH) criteria were met there was a high suspicion of HLH: fever, splenomegaly, pancytopenia, hyperferritinemia, hypofibrinogenemia (0.9 g/L; ref: 1.3–3.3 g/L), and hypertriglyceridemia (4.63 mmol/L; ref: 0.3–1.3 mmol/L) and increased levels of soluble interleukin-2 receptor (10 646 U/mL; ref: <555 U/mL) [2].

An underlying CMV or EBV infection was ruled out by serology tests. Immunophenotyping on peripheral blood was not indicative of a hematologic malignancy. Because the patient had traveled to Morocco, visceral leishmaniasis (VL) was considered as an underlying disease.

Immunophenotyping by flow cytometry on bone marrow aspiration was also normal, and no signs of hemophagocytosis were seen in this sample. However, oval nucleated amastigotes, known as Leishman-Donovan bodies, were detected and led to the diagnosis of VL (Figure 2). *Leishmania infantum* species were confirmed by bone marrow PCR. In addition, PCR of blood was positive for *Leishmania* species as well. Consequently, treatment with intravenous liposomal amphotericin B (4 mg/kg/day) for 10 days was initiated.

**Figure 2.**
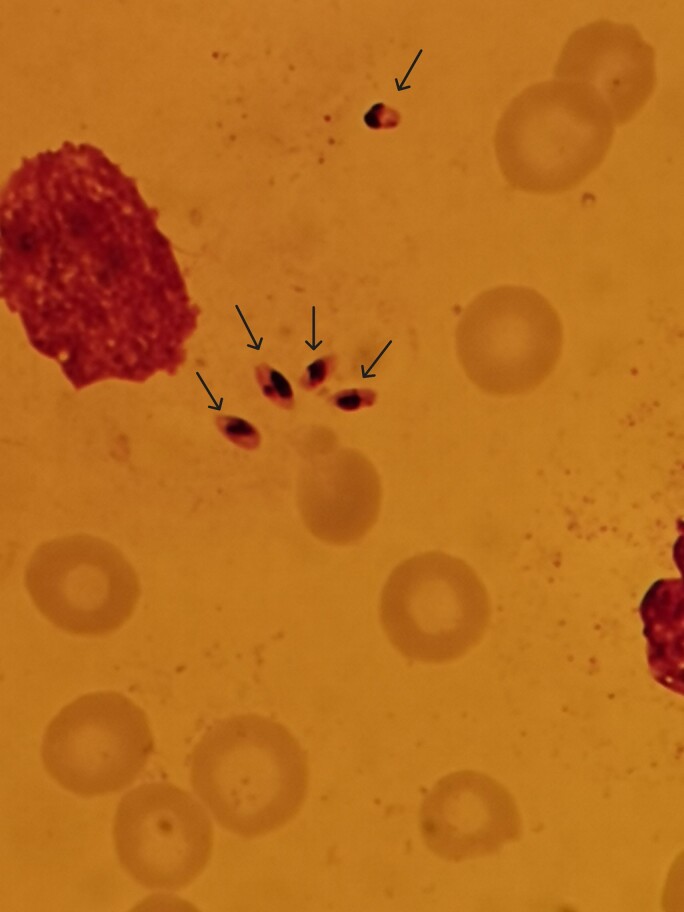
Bone marrow biopsy microscopy revealing Leishman-Donovan bodies (arrows).

The patient showed clinical improvement initially, with a resolution of fever within 24 hours. Unfortunately, fever recurred accompanied by deterioration of pancytopenia and inflammatory markers. Therefore, ongoing HLH was considered. This phenomenon has been described previously [3, 4], but evidence of the benefits of additional HLH treatment in these specific cases is scarce. The deteriorating situation of the patient while on adequate VL treatment led to the decision to start immunosuppressive therapy consisting of prednisolone (2 mg/kg) for 5 days. Treatment with liposomal amphotericin B was continued, and this combined therapy resulted in the resolution of fever, as well as gradual normalization of the pancytopenia and inflammatory markers. Further recovery has been unremarkable.

Leishmaniasis is a vector-borne disease and parasites are transmitted through the bite of infected female phlebotomine sandflies. Visceral leishmaniasis is the most severe form of human leishmaniasis and potentially life-threatening. Each year, an estimated 50 000–90 000 new cases of VL occur worldwide. Age distribution varies considerably per region and per year, but many cases also concern (young) children [5, 6]. The most endemic regions are East African countries, Brazil, and India. Depending on geographic location, VL is mainly caused by *Leishmania donovani* and *L. infantum*, and the main reservoir hosts are humans and dogs, respectively [7]. The disease is characterized by irregular fever, hepatosplenomegaly, anemia or pancytopenia, and weight loss [5]. Treatment of VL consists of antileishmanial therapy. Liposomal amphotericin B is the first drug of choice, if available [8].

Hemophagocytic lymphohistiocytosis is a rare and severe systemic inflammatory syndrome with several causes. Primary (genetic) HLH is usually an autosomal recessive disorder and therefore in our case less likely because of the non-consanguineous parents. Secondary HLH can be triggered by viral, bacterial, fungal, and parasitic infections, as well as neoplastic and autoimmune diseases. Hemophagocytic lymphohistiocytosis is characterized by uncontrolled immune activation causing tissue damage and can become fatal due to multiorgan failure when left untreated. In case of a mild clinical picture, treatment of the underlying disease can be sufficient to terminate the hyperinflammatory process. In critically ill children first-line induction treatment consists of dexamethasone and etoposide and should be initiated immediately upon admission, in addition to treatment for the underlying condition [2, 9].

Characteristics of VL can mimic HLH, but VL is also a potential trigger of secondary HLH, so its occurrence should be in a clinician’s differentials. Although it is rare, there are endemic countries where VL-induced HLH is reported in up to 15% of all pediatric VL cases [4]. Treatment of the underlying leishmania infection is often sufficient for HLH to resolve [3, 4]. However, there are reports of VL-associated HLH in which VL treatment only did not result in clinical recovery. These patients may benefit from additional HLH treatment consisting of immunosuppressive therapy [4]. In our case we observed clinical improvement by adding steroids only.

Teaching points:

- Classic manifestations of VL are fever, hepatosplenomegaly, and pancytopenia. These clinical characteristics overlap with HLH.
- Bone marrow biopsy microscopy is recommended to detect Leishman-Donovan bodies. *Leishmania* species can also be detected by PCR of bone marrow or blood.
- Whenever a patient deteriorates despite adequate antimonial treatment, VL-associated HLH should be (re)considered.
- Treatment of VL only in VL-associated HLH can effectively resolve HLH in most cases; however, in some cases, additional immunosuppressive treatment is needed.

## Notes

Written informed consent for publication was obtained from the parent.

***Acknowledgments.*** I. V., S. A. S. E. K., and J. A. v. d. M. had the lead in writing the manuscript as medical students and contributed equally. S. E. performed the microscopy of the bone marrow biopsy and provided Figure 1. C. V., E. v. N., and M. S. W. Q. were all involved in the clinical care of the described patient. All authors contributed to drafting/revision of the manuscript for content. The Erasmus MC University Medical Center has a government-recognized expert center for leishmaniasis.
